# Humanizing processes after harm part 1: patient safety incident investigations, litigation and the experiences of those affected

**DOI:** 10.3389/frhs.2024.1473256

**Published:** 2025-01-03

**Authors:** Lauren Ramsey, Laura Sheard, Justin Waring, Siobhan McHugh, Ruth Simms-Ellis, Gemma Louch, Katherine Ludwin, Jane K. O’Hara

**Affiliations:** ^1^ Yorkshire and Humber Patient Safety Research Collaboration, Bradford Institute for Health Research, Bradford, United Kingdom; ^2^ York Trials Unit, University of York, York, United Kingdom; ^3^ Health Services Management Centre, University of Birmingham, Birmingham, United Kingdom; ^4^ School of Humanities and Social Sciences, Leeds Beckett University, Leeds, United Kingdom; ^5^ School of Psychology, University of Leeds, Leeds, United Kingdom; ^6^ School of Healthcare, University of Leeds, Leeds, United Kingdom; ^7^ Research and Innovation, Midlands Partnership NHS Foundation Trust, Stafford, United Kingdom

**Keywords:** patient safety, patient involvement, staff involvement, healthcare harm, safety investigations, healthcare litigation, qualitative research

## Abstract

**Background:**

There is a growing international policy focus on involving those affected by healthcare safety incidents, in subsequent investigations. Nonetheless, there remains little UK-based evidence exploring how this relates to the experiences of those affected over time, including the factors influencing decisions to litigate.

**Aims:**

We aimed to explore the experiences of patients, families, staff and legal representatives affected by safety incidents over time, and the factors influencing decisions to litigate.

**Methods:**

Participants were purposively recruited via (i) communication from four NHS hospital Trusts or an independent national investigator in England, (ii) relevant charitable organizations, (iii) social media, and (iv) word of mouth to take part in a qualitative semi-structured interview study. Data were analyzed using an inductive reflexive thematic approach.

**Findings:**

42 people with personal or professional experience of safety incident investigations participated, comprising patients and families (*n* = 18), healthcare staff (*n* = 7), legal staff (*n* = 1), and investigators (*n* = 16). Patients and families started investigation processes with cautious hope, but over time, came to realize that they lacked power, knowledge, and support to navigate the system, made clear in awaited investigation reports. Systemic fear of litigation not only failed to meet the needs of those affected, but also inadvertently led to some pursuing litigation. Staff had parallel experiences of exclusion, lacking support and feeling left with an incomplete narrative. Importantly, investigating was often perceived as a lonely, invisible and undervalued role involving skilled “work” with limited training, resources, and infrastructure. Ultimately, elusive “organizational agendas” were prioritized above the needs of all affected.

**Conclusions:**

Incident investigations fail to acknowledge and address emotional distress experienced by all affected, resulting in compounded harm. To address this, we propose five key recommendations, to: (1) prioritize the needs of those affected by incidents, (2) overcome culturally engrained fears of litigation to re-humanize processes and reduce rates of unnecessary litigation, (3) recognize and value the emotionally laborious and skilled work of investigators (4) inform and support those affected, (5) proceed in ways that recognize and seek to reduce social inequities.

## 1 Introduction

Identifying and investigating safety incidents has been a longstanding, ubiquitous and global focus for the field of patient safety [e.g., (1, 2)]. In the English National Health Service (NHS), these activities are most recently governed by the Patient Safety Incident Response Framework (PSIRF) (3, 4), replacing the previous Serious Incident Framework (SIF) both published by NHS England (5). Both national policy directives have mandated the involvement of those affected by incidents within investigations for almost a decade but have not yet appeared to have translated into routine practice locally [e.g., (6–8)]. This is despite a lack of involvement being repeatedly blamed for a history of well-documented care failings across settings [e.g., (9, 10)], and a growing body of evidence highlighting multiple key reasons to meaningfully involve those affected by incident investigations.

One reason to involve those affected in incident investigations is that it meets a democratic consumer right, and speaks to a restorative view of justice, whereby genuine attempts to rebuild trusting relationships are central (11–13). Evidence suggests that patients and their relatives, as well as staff, report physical, financial and/or emotional vulnerability following healthcare incidents (14–17) and during investigations (18, 19). Morrison et al., described investigations as a “painful journey; for most, a pain yet to heal” (20), resulting in outcomes such as poorer health, work absenteeism and difficulties contributing to society (21, 22). It has been argued that supporting those affected in these circumstances is a system-wide responsibility (23). This field of thought has been increasingly recognized over the past two decades in the UK, albeit slowly. For instance, the Being Open Framework (24) was launched, followed the right to an apology, support through complaints of poor quality or unsafe care, and the commitment to learn and improve services being written into the NHS Constitution (25). The professional Duty of Candour was also legislated in 2014 [Health and Care Act (26)] and NHS England launched the Just Culture guide (2018) in more recent years. Nonetheless, a report from the former Healthcare Safety Investigation Branch (27) highlighted that staff continue to lack support following incidents, and Cribb, O’Hara and Waring (11) call for a more sophisticated understanding of “justice” and justice for whom, in these circumstances.

A second reason—sometimes called the technocratic rationale—to involve those affected in incident investigations is that it enables the healthcare service to learn from their valuable perspectives (13). Since the early call from Vincent and Coulter (28) for an active role for patients in patient safety, research has established that they and their families are a key source of often untapped information that could help support the monitoring, measuring and management of healthcare safety (29–34). The technocratic rationale for involvement underpins the introduction of recent initiatives, such as Martha's Rule—a patient safety initiative in the NHS that gives patients, families, carers, and staff access to a rapid review from a separate care team at any time (35). Nevertheless, despite a growing emphasis on stakeholder involvement, there remains a surprising lack of evidence to support the effectiveness or impact of recommendations resulting from investigations (36).

Other, under-researched considerations are reasons for pursuing litigation. There are an estimated 11,000 reported incidents causing severe harm or death in the UK annually (37), resulting in approximately £1.7billion worth of clinical negligence claims, with a further £1.8billion to administer and settle claims, and long-term liabilities amounting to £65billion (38). The literature is also inconsistent as Anderson, Allan and Finucane (39) found no link between complaints and litigation, and suggestions that legal implications are a key barrier to participation (40), yet NHS Resolution (41) proposed that some claims were driven by frustration and poor experiences of investigation processes, suggesting that involving patients and families earlier would see reduced complaints (41).

In summary, there is growing interest in involving those affected in investigations of incidents, yet there remains little UK-based empirical evidence exploring the experiences of key stakeholders over time, including factors influencing decisions to litigate. Therefore, we sought to explore the following research questions: (i) What is the experience of patients, their families, healthcare staff, investigators and legal staff who have been involved in an incident investigation over time? (ii) What might influence the patient or families decisions to litigate?

## 2 Methods

This interview study received favorable ethical opinion in July 2020 (REC Ref—20/EE/0133). Interviews took place between Sept 2020 and April 2021.

### 2.1 Recruitment

A targeted sampling approach aimed to recruit participants via: (i) a personal invitation letter, general communication method or snowball sampling at four NHS Trusts and an independent investigatory body in England (ii) advertisement via relevant charitable organizations (Care Opinion www.careopinion.org.uk/, AvMa www.avma.org.uk/, Harmed Patients Alliance harmedpatientsalliance.org.uk/), (iii) advertisement via social media, or iv) word of mouth. The method of recruitment was purposive and directed towards patients, relatives and staff who had been involved in safety incidents and subsequent investigations. People registered their interest by email, were provided with a detailed information sheet (in easy-read when preferred) and were assessed for eligibility via telephone. Criteria stipulated that participants must: be >16 years old, have experienced a “serious incident” and subsequent investigation within a healthcare setting as defined by the Serious Incident Framework (5), experienced the serious incident >1 year after consenting to take part, have no related ongoing police or legal involvement relating to the incident, and have capacity to consent. Eligibility assessment followed a detailed semi-structured guide. Participants were signposted to personalized sources of support where necessary.

**Figure 1:**
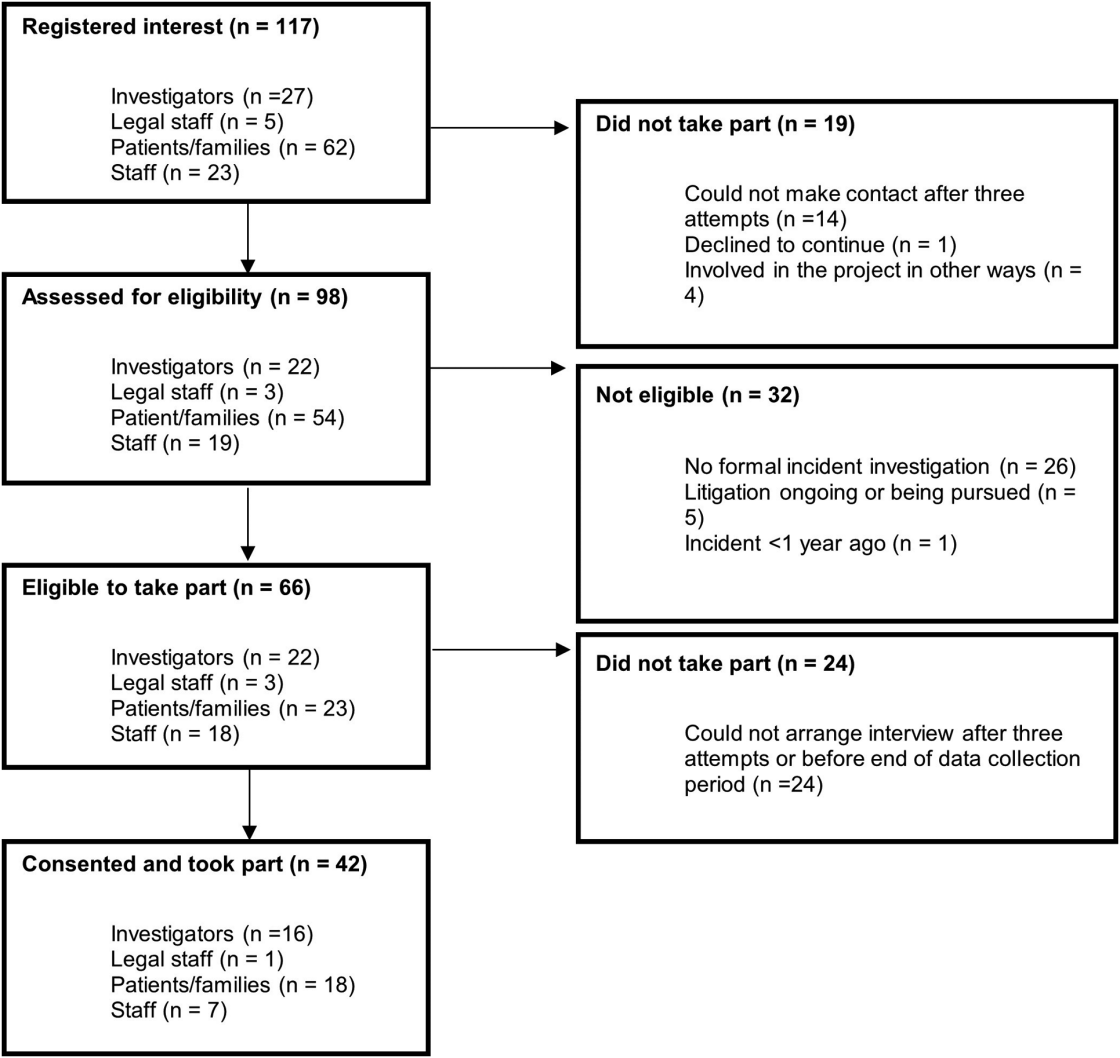
Participant flow.

117 people registered interest, 98 people were assessed for eligibility and 66 were eligible, of which 42 consented to participate (see Figure 1). Decision to stop recruitment was made based on collaboratively feeling that we had “understood enough” and “heard enough” (42), as well as being guided ethically, wanting to avoid unnecessarily causing distress to those not eligible, consenting participants and the research team. Specific demographic data was not collected from participants.

### 2.2 Interviews

Interviews followed a topic guide which enabled avenues of conversation to remain focused on the research questions, while also allowing flexibility to capture wider topics of interest, including exploring topics most important to participants themselves. Topic guides were tailored for each stakeholder group, however, all questions centered on experiences of incident investigation processes, their thoughts and feelings about, and experiences of, involvement, and their experiences of interlinked processes including decisions to litigate. The topic guide was developed based on the focus of the research questions, the exploratory nature of the study and also to reflect existing research findings from the wider programme of work [e.g., (6, 18)]. With guidance and support, participants were given the option of producing and sharing a timeline to organize and structure their thoughts and ensure that they were able to share details of events that were most important to them personally, as well as helping the researcher to understand the order of events during the interview (43). However, timeline data were not included in the analysis.

Interviews took place virtually due to COVID-19 restrictions, held via Zoom (*n* = 36), Teams (*n* = 2) or telephone (*n* = 12) and were video and/or audio recorded. Interview duration ranged from 25 min to 2 h 32 min (average 1 h 27 min). Researchers followed a detailed distress protocol aiming to support both participants (e.g., via signposting to tailored support) and researchers (e.g., debriefing after each interview), led by a researcher with a background in counselling (RSE).

### 2.3 Analysis

Data were auto-transcribed via Zoom or Teams software initially where possible and corrected, or transcribed. An inductive reflexive thematic approach was taken to analysis (44), aiming to develop overall findings representing the commonality of experience across the stakeholder groups, and explore divergence. Weekly “data sessions” were held with interviewing researchers to reflexively discuss initial impressions, and develop a descriptive account based on patterns of meaning and similarities and differences within and between participants and stakeholder viewpoints. Researchers then aimed to elucidate the descriptive accounts, and ensure they were grounded within the data. This involved reading each transcript to become immersed within the data, and making descriptive notes in the margins, as well as highlighting significant quotes and summarizing key details of each account, before independently and collaboratively collating ideas analytically. Further discussions were held with the wider research team, including qualitative experts (LS, JW), a patient and family advisory group and a staff advisory group, to develop, evidence and refine the themes until a consensus was reached.

### 2.4 Findings

Forty-two eligible people with lived or professional experience of incident investigations took part in individual semi-structured interviews with one of four researchers (LR, KL, RSE, SMcH). The 42 interviewees included seven patients directly affected by the incident and twelve relatives. Thirteen of those experience related predominantly to acute care and five related to mental health care, although some spanned multiple settings. Two others related to separate investigations or inquiries and one was completed by an independent investigatory body rather than a local Trust. Incidents included six delayed/misdiagnoses, four surgical errors, two maternity harms, three suicides, two unexplained deaths and one drug error. Five healthcare staff and three investigators worked within acute care and two healthcare staff and three investigations worked within mental healthcare settings. Five investigators worked within national settings and one worked across settings as a bank investigator. One legal staff took part. We constructed five themes that reflect and address the research questions.

### 2.5 Cautious hope colliding with fear

Patients and families described vulnerability, largely emotionally, following harm while under the care of a service they inherently trusted.

“I was very distressed. I find myself quite a resilient person. I can manage my emotions quite well. But I think I was very, very vulnerable in that situation.” Patient

Due to the range and complexity of emotions that some experienced while coming to terms with the immediate and longer-term implications of what had happened, patients and families struggled to know what to do, particularly in the absence of support.

“When you’re low like that you don’t know what to do, you don’t know how to raise issues, you don’t know where to go… To start with I did nothing. I was just like, completely dumbfounded.” Patient

Having never experienced an incident before, some described difficulties disentangling their experiences of care and what happened next, as well as feeling overwhelmed by opaque and unfathomable processes. This became more complex when interrelated processes ran alongside investigations, with overlapping timelines and unclear remit (e.g., coroner's inquest, police investigations, patient advice and liaison services, formal complaints, funeral planning, litigation, public inquiries, and independent investigations).

“We were getting drip-fed information, and because there were so many different agencies sort of involved… the whole thing’s really, really difficult. Really difficult… I just don’t think that anyone has sort of, really helped us at all.” Relative

However, most proceeded with cautious hope. Caution was fueled by the incident itself, as well as perceptions of delayed or disregarded escalation attempts and histories of fractious relationships and poor communication with care teams. Hope, on the other hand, was fueled by reassurance at the existence of investigation processes bringing an opportunity to be heard, and wanting to feel able to trust the healthcare service again for some good to come of what happened.

“During the time of my mum being looked after, she was not listened to and also as a family we weren’t listened to, so it felt like an opportunity, finally, to be listened to.” Relative

Most described placing good faith in the system, with expectations of being proactively supported with empathy and compassion. How that good faith was handled then laid the foundations for ongoing relations. Over time, a sense of injustice was fostered, not only by what happened (e.g., jarring and defensive tones to communication), but also what did not happen (e.g., apology or offers of support).

“We didn’t realize that we were going to be met with such hostile feelings. We thought everyone would have wanted to get to the bottom of what happened… We didn’t realize it was all going to become a, well I don’t know, just, ‘let’s sweep it under the carpet,’ because nobody wanted to be blamed.” Relative

This was perceived to be largely driven by fear of legal repercussions, seen as a barrier to rebuilding trust, transparency and learning.

“There’s a lot of fear in involving the family or apologizing because it gets confused with some sort of admission of blame or liability.” Investigator

This was a fear that was deemed culturally engrained and difficult to overcome.

“I think it’s difficult to, you know, as a Trust [to] put yourself in the path of inviting litigation.” Staff

For staff, investigations invoked shame, blame and fear of what that meant for the patient, as well as their reputation and the security of their professional position. Often continuing to work clinically in the immediate aftermath, staff described a lack of time and mental space to process their experience. Other times, staff were made formally aware of an investigation by management, sometimes perceived to be insensitive, unsupportive and questioning of their professional capabilities, having lasting impacts on them, or learned of investigations informally via colleagues or patients.

“It was almost a case of, the nursing staff, you know, had almost been told, oh, just be careful of this one. So, it felt very isolating… I felt very scrutinized.” Staff

### 2.6 On the side lines of organisational agendas

Over time, patients and families experienced widening power gaps, leaving them disillusioned by a lack of compassion, acknowledgement and accountability. Some felt that they had to present in an emotionless manner to be perceived reasonable resulting in further breakdown of relationships. Muddling through a complex system with limited knowledge, resource and power, designed without their needs in mind meant that everything became a challenge at a time they needed support.

“I didn't have the information or knowledge to explain exactly, and my eyes started filling up… when you know inherently there’s something wrong because you can hear enough information, but you can't join all the dots, nobody’s joining the dots for you.” Patient

In the meantime, some blamed themselves for the outcome or felt made to question their own memories and realities, leaving them more vulnerable.

“You begin to believe that you’re making it up somehow. I remember that at one point thinking ‘well if no-one’s believing me, it must be my fault’ and of course it wasn’t, and even if it was then I needed help, you know.” Patient

Some persisted. Others with limited power, strength, assertiveness, systems intelligence, and social capital withdrew. Legal involvement was also a barrier to rebuilding relationships, as communication went through additional layers of scrutiny and demanded the involvement of different personnel.

"I think it’s sad because if there are solicitors for the family, I think the Trust are obliged to have our Trust solicitors… it’s escalated to a different level and makes it less personal.” Investigator

Staff experienced parallel isolation, sometimes separated from the patient and family they cared for going against naturally caring instincts to reach out to them. This distancing was thought to be designed to protect staff, but inadvertently fostered unresolved feelings, guilt and apprehensions of unknown outcomes, as well as fear of how events may unfold if they came into contact informally. Generally, there was a sense that processes were designed to protect the organization, over and above learning or supporting those affected.

“The terror that the thought of an investigation just still instils in staff… I recall being investigated… like I've seen it from different angles, erm, and I'm very aware of how people feel… I had been a midwife for ten plus years.” Investigator

Concerns surrounded the combination of accountability and ambiguity, as well as perception that the system was unfair, biased and purposefully excluded them.

“It felt very sneaky… I was told to my face ‘now, this is all about transparency and getting to the bottom of what happened, and improving patient safety’ and all the rest of it, and then, when I saw the report it was abundantly clear that’s exactly not what was happening.” Staff

For some, seeking support was considered an unnecessary indulgence. Staff described the longer-term impacts this had on their well-being and job satisfaction, contributing to decisions for some to avoid working in certain settings or ultimately leave their role.

“It was really difficult for me because it wasn't a case of this is in your best interests or whatever, it was more of it’s in the best interests of your employers, the Trust, or my manager.” Staff

Investigators also had complex social identities, entangled with personal beliefs and the motivations of patients, families and staff, as well as organisational pressures from legal teams, governance structures, and wider local and national policy directives.

“I can hear from the family. I can hear from the staff… You don't have an allegiance to either camp. So, holding both camps, is what we have to do, in my view, what you have to do if you're a compassionate investigator… It is exhausting. I have a view that actually a better model, is why doesn’t our families have an independent advocate of their own?… Then I know the family is properly looked after and I can get on with my job without feeling pulled. And it’s not that I won't have a relationship with the family. I think it just puts us a little buffer zone there.” Investigator

Factors including their background (e.g., some investigators were current or ex-clinical staff), power (e.g., influence of senior or legal teams), morality (i.e., what felt like the right thing to do) and organizational culture (i.e., the way things tended to be done), shaped their approach.

“The culture of how we treat families comes from the top.” Investigator

Some were keen to distance themselves from “organizational agendas”, experiencing isolating internal conflict as a result.

“It’s quite an isolated role, you know, I'm not part of the team. I mean, technically I am… but even before Covid I would be working at home for most of the time. I like that distance.” Investigator

Additional challenge included feeling inadequately equipped with skills and knowledge in working sensitively and meaningfully with those affected by safety incidents, as well as investigating alongside a demanding clinical role.

“I’ve always really struggled with erm, I wouldn’t say the lack of support we provide to families, but how it’s been quite difficult in my role, because by default, there’s nothing else. I almost become that kind of support or signposting for families. And that’s something I feel really strongly about… I get sworn at a lot… it’s never going to be a great, you can’t make this process sound like a nice day out, because there’s tragedy involved.” Investigator

However, the independent investigatory body had relatively well-established processes, in which investigating was their only role, and with organizational investment in training and support, of which most spoke positively about. Nonetheless, across contexts, being the human face following healthcare harm was emotionally laborious, with risk of burnout and suggestions that the role was potentially unsustainable long term.

“I don’t think anyone should do this job longer than three or four years… there is a chance that people will become emotionally burnt out… it’s certainly a job that, you know, you wouldn’t want to still be here ten years down the line dealing with these sorts of incidents.” Investigator

Some felt unsupported in their role, with no outlet for the emotional toll the role took on them. The combination of emotional labour and lacking protected time in their job plan, meant that some investigators did not reach out to patients and families as much as they would have liked to.

“People are trying to do it when they've got a spare hour.” Investigator

### 2.7 Awaited, yet unwelcome report

Investigators were tasked with producing a coherent narrative report, despite messy realities, conflicting accounts and gaps in their understanding of what happened. Some investigators were also frustrated at the repetitious and inevitable nature of investigations, and lacking confidence that recommendations would be implemented.

“Then the family always ask me, how do we guarantee that they are going to act on the findings, that is literally the first question. And that’s quite a tricky one to answer because we don't have any empowerment to be able to do that… in all reality, a Trust could take your report and put it in a drawer and it never see the light of day again… they want their baby’s story to make a difference and make that service safer. So, it is quite difficult, as an investigator, for me to be able to then say, well, actually I have zero control of what they do with that.” Investigator

Patients and families waited in anticipation of the report, marking a key point in the process at which divergent perspectives came together. Reports were often described as disheartening, disrespectful, dishonest, paying lip service, defensive, lacking empathy, and avoiding accountability.

"I can safely say—I’ve been around for a while now, and in all those years I've never ever seen my mum more angry or upset than the day I turned up at her house to see her reading through that report.” Relative

Some also felt that the report insensitively delivered unexpected information, indicated ambivalence and that the organization had lost sight of the affected family, devaluing their experience and disregarding the questions they had raised.

“The report doesn't really even sort of acknowledge the fact that she died… Small things like typos which maybe in another context you would let go… at various points they refer to her as Mr… in this context feels just really disrespectful.” Relative

Frustration was also felt by patients and families where there was no right to discuss, reply or refine what had been written and it was often considered too late to become meaningfully involved and influence the report in the ways they would have liked to with hindsight.

“I was really trying to be an advocate for this family. It got escalated quite high up, through our head of midwifery into the governance lead for the area. It basically was a blanket, ‘no, that’s the end of the report and that’s it’, and it was awful, it was absolutely awful. Working like that, where the family obviously were not happy and there were things I wanted changing.” Investigator

Following receipt of the report, some were offered bereavement support, funeral planning advice, counselling service access, and signposted to relevant charities. Others were invited to meet with representation from the healthcare organization to ask any outstanding questions. However, for some, offers of support were absent, inappropriately timed, or considered tokenistic or inaccessible. Instead, some sought to build informal relationships with others who had experienced similar situations and developed their own support networks.

“We were given a booklet with various charitable organizations to ring… I actually had a friend, her husband had taken his own life. And she'd rung one of these organizations on the list and said she got someone at the end of the phone who was not very warm. And she didn't think it was particularly helpful… sadly I know, two or three people that, their husbands have taken their own lives, and so I've had support from them more than anything… I think we could have done with some more help… the fallout that it’s had on the family as a whole has been enormous. It’s totally changed our lives. In some ways the shock in the early days you can't, you're not open to help.” Relative

### 2.8 Left with an incomplete narrative

Once reports were published, there was a general sense that it was the end of the organisational process, leaving those affected—including staff, to pick up the pieces, sometimes with their life sometimes in turmoil.

“I had such a terrible experience that really scarred me, to the point of wondering for a while, whether I still wanted to be a doctor… I hated going to work, I absolutely dreaded it. I love my job, but I hated it with a passion… not one person at any point asked me if I was okay… I'm lucky to have friends and family… but my God, they could have ended up with a very, very different outcome, had I not had support. And that really angers me… It’s really important that we don't traumatize already traumatized staff further.” Staff

Patients and families experienced a heightened sense of fear, confusion and disorientation, continuing to live with profound personal impacts, as well as having concerns for the mental toll it took on those around them. For some, life became an all-consuming effort to get answers to their questions and help to prevent the same thing happening to others, which was described as an exhausting, emotional and lonely journey that continued to have a ripple effect on their life.

“I had to sort of step back and take a breath, and when I looked around me after all the years I'd spent… intensely engaged in this, but the rest of my life crumbled around me… it broke me, if I’m honest.” Relative

People also reflected on being drip-fed information, leading to more questions they felt compelled to gain answers to via activities such as liaising with clinical experts, regulatory bodies and members of parliament, as well as sourcing relevant information including recent reports and policy linked to the incident.

"I've had cause to touch base with the police, with home office pathologists, with countless regulators… I have spent hundreds of hours watching live surgical procedures… I reckon I could take a decent stab at performing this procedure… We are kind of forced into the level of detail that no normal person outside of medicine would ever have to get involved in, but you're forced down that route in order to understand.” Relative

The sense that opportunities to learn were neglected also contributed to compounded harm, as some felt that their experience was of no consequence. Some perceived that this was due to procedural constraints, whereas others evaluated that the organizations chose to circumvent the real issues that needed attention. Others raised concerns of arbitrary recommendations.

“It makes it feel that what you’ve gone through hasn’t been completely in vain… I just feel that yes I went through this and anyone else can go through it again afterwards, it’s, you know, no-one’s learnt anything from it.” Patient

### 2.9 Litigation as a last resort

Pursuing litigation was often not financially motivated, or a decision taken lightly, but was considered an avenue people were forced down in hope to be finally heard, gain answers to their questions, and receive some recognition for what had happened and its impacts.

“When I did, you know, seek legal advice, that wasn't something that was a small decision, it was a massive decision. I just felt like it was the only way. I wanted to have a proper investigation and I just wanted them to take notice.” Patient

Some felt that outside of litigation, there was nobody with the power needed to help them piece together the puzzle. Others reflected on what was perceived unnecessary distress, having to go through the legal process, and that the organisation could have taken simple steps to avoid forcing them down that route.

“If this was a mistake that had been immediately acknowledged and admitted to in the spirit of the Duty of Candour as it should be, this probably wouldn't have even been a clinical negligence claim. Don’t get me wrong, we’d have been a bit p’ed off but we’d have got past it, you know, we recognize that, hey, we're all human.” Relative

On gaining legal advice, some found the unhindered communication a refreshing contrast to a process that was seemingly out to question their realities, meaning it was the first time they felt heard.

“That was the first time that somebody had just listened and then taken it all in… that validation just helps you.” Relative

There was however, an acknowledgement that pursuing litigation required capital, both financially and mentally, to allow people to repeatedly revisit what happened, whilst also suffering potentially life changing consequences, or grieving. This sense of powerlessness led some to choose not to raise a legal claim.

“At one stage I was so upset by the whole thing I felt like taking legal action but I was very aware that the NHS is a very large organization and, you know, that it was little me against them. I didn't feel like I wanted to take it on… I felt like I was in the boxing ring with my hands tied behind my back. And I felt desperate.” Relative

Concerns were also raised for others who may be defeated by the process due to social inequity.

“It discriminates against people who don't necessarily have the ability or the support around them to pursue it… or have the intelligence and have the drive to go through the compensation process.” Patient

## 3 Discussion

The study aimed to explore the experiences of patients, families, staff and legal representatives affected by safety incidents over time, and the factors influencing decisions to litigate. In doing so, we found that patient safety incidents meant that patients and families went on a journey, including various stages of hope, disappointment and significant impact on their lives. Patients and families started investigation processes from a point of cautious hope, expecting the health service to want to listen and learn from what happened, as well as support them to heal. However, later, they came to realize that they lacked power, knowledge, and support to navigate the system, with risks of disproportionately affecting those most vulnerable. Some felt intentionally excluded, illuminated in awaited investigation reports. Overall, there was a sense that elusive “organizational needs” were prioritized above the needs of all affected. As a result, some felt forced to meet their needs elsewhere, such as pursuing litigation. Ultimately, emotional distress was experienced by everyone involved, yet processes neglected emotion, resulting in compounded harm. Our findings illuminated a parallel journey experienced by staff, who also faced a series of challenges and sometimes disappointments.

Just like healthcare staff, patients and families are emotional beings (45). The findings from this study highlight the undoubtable importance, impact and scale of emotional harm experienced by everyone affected, and failure to recognize and address such, contributing to compounded harm (46). Acknowledging that types of compounded harm center on emotion being imperative, as emotional harm is often ignored, or its importance minimized in favor of physical, and to some extent's financial reparation (7). As a result, we recommend that investigatory processes should be relational, centering the needs (including emotional) of patients, relatives and staff affected by safety incidents to avoid compounding harm (see Box 1). Innovative restorative approaches are being adopted in other nations such as New Zealand (47, 48). A separate, secondary analysis of this data also focusses specifically on the types of compounded harm experienced by patients and their families as a result of responsive processes—powerless, inconsequential, manipulated, abandoned, de-humanized, and disoriented (49) Designed in part, to support the emotional needs of patients and families, is also a role that is being increasingly established in the English healthcare system—the Patient Family Liaison Officer (PFLO) (Overton et al., In Prep), leaning from initiatives better established in policing. Nonetheless, research into such approaches remain in their infancy, and organizational readiness for them is yet to be empirically explored within the context of the NHS.

BOX 1 **Recommendations.**

**In light of the findings, we propose five recommendations:**

1) Investigatory processes should be relational, centering the needs (including emotional) of patients, relatives and staff affected by safety incidents to avoid compounding harm.

2) The relational work carried out by investigators is both important and complex, and needs to be adequately resourced, valued, and recognized within policy and processes.

3) Patients, relatives and staff should have access to tailored information to aid their understanding of what investigation processes entail, how they can become meaningfully involved, and how they can effectively navigate the system in flexible ways.

4) Investigation processes should recognize and seek to reduce social inequities, providing tailored support to those who need it.

5) Policy and procedure directives at local and national level, as well as support from outside agencies (e.g., regulatory bodies) should be in alignment to overcome culturally engrained fears of litigation at all levels of the healthcare system.

Interestingly however, recognition of the centrality of emotion in investigatory processes is not new, with authors two decades ago suggesting a need to “go back to basics in healthcare” and dignify these very human experiences, with a human response (50). What our study does help to shed light on, is why what may seem both morally and logically the right thing to do (51), may be more complex to deliver in reality, than it appears from any individual perspective. To our knowledge, this represents the first UK-based study to examine the views of key stakeholders collectively—patients, their families, healthcare staff, investigators and legal staff, and over time. Our findings extend current literature by exploring both the convergence and divergence in the experiences of these important groups of people, with evidence of significant synergies, in particular between patients, relatives, and staff. Indeed, whilst traditional narratives of investigations might posit staff and patients and families as adversaries in investigations, we found compelling evidence that patients, relatives and staff often reported similarly feeling overwhelmed, excluded, ill-equipped, unsupported and uninformed. This contribution is significant, as current guidance and practical frameworks guiding involvement in investigations are often based on data from one perspective (19, 52), or developed within other healthcare economies (53–55).

In addition, an important finding of our research is that investigators felt their role was largely invisible and undervalued, leading to feeling isolated and at risk of burnout with limited knowledge, training and support. Investigators may shoulder unmanageable responsibility to navigate the balancing act of completing organizational priorities and sensitive discussion with those affected. As a result, we recommend that the relational work carried out by investigators is both important and complex, and needs to be adequately resourced, valued, and recognized within policy and processes (see Box 1). Implementing PFLO's may help to alleviate such pressure, or indeed, may shift or event amplify the pressure with heightened expectations. Further research is needed to determine the acceptability and feasibility of such role, including unintended consequences.

As well as the emotional harm, those affected also felt excluded from processes, and struggled to become meaningfully involved in a system that did not welcome them, support them or help them to understand. As a result, we recommend that patients, relatives and staff should have access to tailored information to aid their understanding of what investigation processes entail, how they can become meaningfully involved, and how they can effectively navigate the system in flexible ways (see Box 1). An important lens to look at these findings, is that of epistemic injustice (56), which has previously been explored by Kok et al. (57), within the context of incident investigations in Dutch health services. We recommend that investigation processes should recognize and seek to reduce social inequities, providing tailored support to those who need it. However, further research is needed to explore what that might look like, and whom might best benefit.

Perhaps even more complex to change it the wider cultural and systemic barriers to re-humanizing processes after harm are driven by organizational fear of litigation. Research from Bell et al. (58), found that patients and families who felt involved in transparent investigation processes were less likely to pursue litigation, whereas others felt the need to fight for progress, using methods such as threatening litigation. To help to overcome these issues, active compensation initiatives have been tested in an American healthcare economy, designed to reduce the burden on patients and families to seek financial recourse while suffering the impacts of what happened. This includes Communication Resolution Programmes (59–62) the Disclosure, Apology and Offer Model (58) and the Recognize, Respond and Resolve initiative (63). Nonetheless, this body of evidence suggests that culturally engrained fears of litigation remain difficult to overcome. Our findings support calls from PHSO (7) that if the healthcare system could be stripped of fear or litigation, and centered the needs of those effected, not only would people experience less compounded harm, but there would also be reduced litigation costs. Without that, there is a risk that no-one's needs are being met, in the pursuit of elusive organisational needs—such as learning, avoiding litigation and managing reputation. Integral to a reorientation of the system, we recommend that policy and procedure directives at local and national level, as well as support from outside agencies (e.g., regulatory bodies) should be in alignment to overcome culturally engrained fears of litigation at all levels of the healthcare system (see Box 1).

### 3.1 Limitations

There are three principal limitations to this study. First, the decision to include legal staff in the study was made part-way through, meaning that we did not recruit as many people bringing this perspective as we would have liked. To help to address this issue, we did however, involve the views of legal staff in the wider steering of the study and programme. Second, the self-selecting nature of participants perhaps attracted those with particularly negative experiences to take part. Third, while we did not specifically collect participant demographic information, we recognize from the findings that equity, diversity and inclusivity was perhaps an issue. Therefore, research is needed to capture experiences of those from a range of demographic groups, and in particular those who might be more vulnerable to patient safety incidents, to ensure the robustness of our findings and conclusions. Research better capturing experiences from those with protected characteristics, for example, would be important to ensure that findings are relevant across healthcare populations and include those potentially most vulnerable.

## 4 Conclusions

Current investigation processes fail to acknowledge and address the emotional harms that are experienced by those affected. As a result, patients and families are experiencing compounded harm and pursuing unnecessary litigation, staff are fearful, investigators are shouldering unmanageable responsibility, and the healthcare organization not visibly learning or improving. To avoid fear-driven processes compounding harm for those affected we propose five recommendations: (1) Investigatory processes should be relational, centering the needs (including emotional) of patients, relatives and staff affected by safety incidents to avoid compounding harm, (2) The relational work carried out by investigators is both important and complex, and needs to be adequately resourced, valued, and recognized within policy and processes, (3) Patients, relatives and staff should have access to tailored information to aid their understanding of what investigation processes entail, how they can become meaningfully involved, and how they can effectively navigate the system in flexible ways, (4) Investigation processes should recognize and see to reduce social inequities, providing tailored support to those who need it and (5) Policy and procedure directives at local and national level, as well as support from outside agencies (e.g., regulatory bodies) should be in alignment to overcome culturally engrained fears of litigation at all levels of the healthcare system.

## Data Availability

The raw data supporting the conclusions of this article will be made available by the authors, without undue reservation.
